# Supplementary material for: PRMT1 Regulates EGFR and Wnt Signaling Pathways and Is a Promising Target for Combinatorial Treatment of Breast Cancer
Source: Cancers (Basel). 2022 Jan 8;14(2):306. doi: 10.3390/cancers14020306 (PMC8774276; doi:10.3390/cancers14020306)
Supplement: Supplementary file 1 [file cancers-14-00306-s001.zip › Supplementary Materials/2022-01-03_Suresh et al._Supplementary information.pdf]

Supplementary information for

PRMT1 regulates EGFR and Wnt signaling pathways and is a promising target for combinatorial treatment of breast cancer

Samyuktha Suresh, Solène Huard, Amélie Brisson, Fariba Némati, Rayan Dakroub, Coralie Poulard, Mengliang Ye, Elise Martel, Cécile Reyes, David C. Silvestre, Didier Meseure, André Nicolas, David Gentien, Hussein Fayyad-Kazan, Muriel Le Romancer, Didier Decaudin, Sergio Roman-Roman and Thierry Dubois

Correspondence to: thierry.dubois@curie.fr

This PDF file includes:

Supplementary Figures. S1 to S21

Other Supplementary Materials for this manuscript include the following:

Table S1 [Antibodies, Primers, siRNAs, and Drugs]
Table S2 [Differentially expressed genes in PRMT1-depleted MDA-MB-468 cells]
Table S3 [PRMT1 DNA copy number gain and loss in the curie cohort]

Figure S1

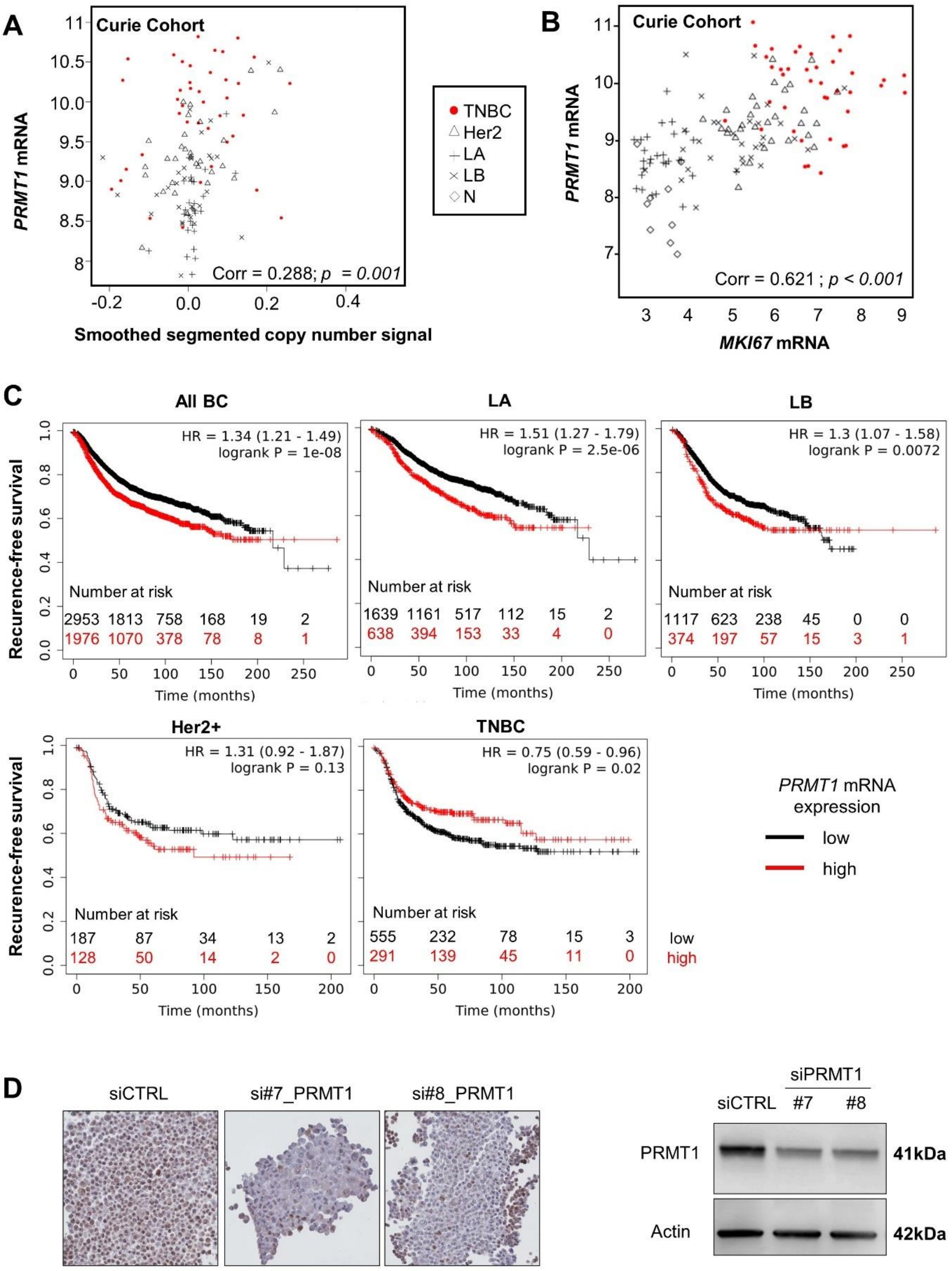

A
Curie Cohort
PRMT1 mRNA
Smoothed segmented copy number signal
Corr = 0.288; p = 0.001
TNBC
Her2
LA
LB
N
B
Curie Cohort
PRMT1 mRNA
MKI67 mRNA
Corr = 0.621 ; p < 0.001
C
All BC
HR = 1.34 (1.21 - 1.49)
logrank P = 1e-08
Number at risk
2953 1813 758 168 19 2
1976 1070 378 78 8 1
LA
HR = 1.51 (1.27 - 1.79)
logrank P = 2.5e-06
Number at risk
1639 1161 517 112 15 2
638 394 153 33 4 0
LB
HR = 1.3 (1.07 - 1.58)
logrank P = 0.0072
Number at risk
1117 623 238 45 0 0
374 197 57 15 3 1
Recurence-free survival
Time (months)
Her2+
HR = 1.31 (0.92 - 1.87)
logrank P = 0.13
Number at risk
187 87 34 13 2
128 50 14 2 0
TNBC
HR = 0.75 (0.59 - 0.96)
logrank P = 0.02
Number at risk
555 232 78 15 3
291 139 45 11 0
low
high
PRMT1 mRNA expression
low
high
D
siCTRL
si#7_PRMT1
si#8_PRMT1
siPRMT1
siCTRL #7 #8
PRMT1
41kDa
Actin
42kDa

Figure S1. Correlation and survival analyses and validation of PRMT1 antibody for IHC. A, *PRMT1* mRNA expression correlates with DNA copy number in the whole population of BC in the Curie cohort (Spearman correlation). **B,** *PRMT1* and *MKI67* (proliferative marker) mRNA expression positively correlate in the whole BC population of the Curie cohort (Spearman correlation). **C,** *PRMT1* mRNA expression correlates with prognosis in BC. Recurrence-free survival based on *PRMT1* mRNA expression (Affy probe ID: 206445_s_at) was obtained from the Kaplan-Meier (KM) plotter website (http://kmplot.com). Best performing cutoff option was used: all BC (n=4929), Luminal B (LB, n=1491), Luminal A (LA, n=2277), Basal for TNBC (TN, n=846), and Her2+ (n=315). Hazard ratio with 95% confidence interval and log-rank p-values were calculated and significance threshold was set at $p < 0.05$. Of note, a similar figure plotting *PRMT1* mRNA expression (median cutoff) vs RFS in the whole BC population has been previously published (Liu et al., 2019, "Methylation of C/EBPα by PRMT1 Inhibits Its Tumor-Suppressive Function in Breast Cancer", *Cancer Res.* DOI: 10.1158/0008-5472.CAN-18-3211) but with a lower number of samples (4929 in our study compared to 3951 in their article). **D,** Validation of PRMT1 antibody for IHC staining. Left panel, PRMT1 antibody used for IHC (Fig. 1C) was validated using AFA-fixed cell pellets from MDA-MB-468 cells treated with control siRNA (CTRL) or two siRNAs targeting PRMT1 for 72 h (#7, #8). Right panel, PRMT1 depletion was verified by western blotting using an anti-PRMT1 antibody. Anti-actin antibody was used as a loading control.

Figure S2

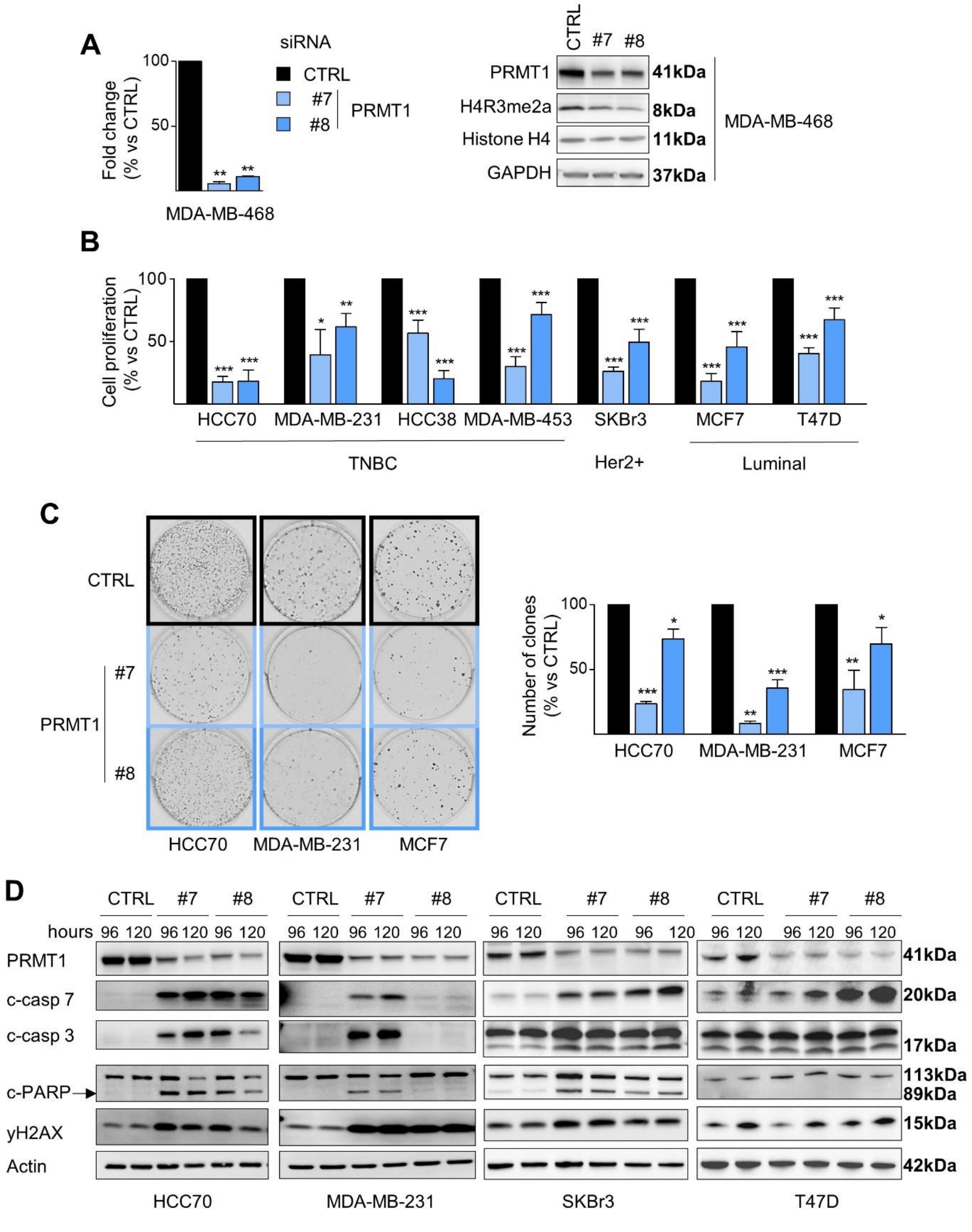
A
siRNA
CTRL
#7
#8
PRMT1
100
50
Fold change (% vs CTRL)
**
MDA-MB-468
CTRL #7 #8
PRMT1 41kDa
H4R3me2a 8kDa
Histone H4 11kDa
GAPDH 37kDa
MDA-MB-468
B
Cell proliferation (% vs CTRL)
100
50
*
**
***
HCC70
MDA-MB-231
HCC38
MDA-MB-453
SKBr3
MCF7
T47D
TNBC
Her2+
Luminal
C
CTRL
PRMT1
#7
#8
HCC70
MDA-MB-231
MCF7
Number of clones (% vs CTRL)
D
hours
96 120
PRMT1
c-casp 7
c-casp 3
c-PARP
yH2AX
Actin
41kDa
20kDa
17kDa
113kDa
89kDa
15kDa
42kDa
HCC70
MDA-MB-231
SKBr3
T47D

Figure S2. PRMT1 depletion decreases cell viability, colony forming ability and induces apoptosis in various BC cell lines.

A, Validation of PRMT1 siRNAs. MDA-MB-468 cells were treated with control (CTRL, black) or two PRMT1 (#7, #8, blue) siRNA for 48h. PRMT1 expression was detected at the mRNA (by RTqPCR, normalization by actin; left panel) and protein (by western blotting, right panel) levels. The methylation of histone H4 on Arg 3 (H4R3me2a) was used to measure PRMT1 activity and anti-histone H4 and anti-GAPDH antibodies were used as loading controls. **B,** PRMT1 depletion decreases the viability of BC cells. TNBC, Her2+, and luminal cells were transfected with control (CTRL, black) or two PRMT1 (#7, #8, blue) siRNAs for 144h and cell viability was measured by an MTT or WST1 assay. **C,** PRMT1 depletion decreases colony formation. TNBC and luminal cells were transfected with control (CTRL, black) or two PRMT1 (#7, #8, blue) siRNAs, and then cultured on plastic for 6 mitotic cycles equivalent to 14 (HCC70), 7 (MDA-MB-231) or 12 (MCF7) days. A representative image (left panel) and the quantifications (right panel) are shown. **D,** PRMT1 depletion induces apoptosis in BC cells. TNBC, Her2+ and luminal cells were transfected with control (CTRL) or two PRMT1 (#7, #8) siRNA for 96h or 120h. Apoptosis was detected by western blotting using antibodies recognizing the cleaved forms of caspase 7 (c-casp 7), caspase 3 (c-casp 3) and PARP (c-PARP). DNA damage was detected using an anti-γH2AX antibody. Anti-actin antibody was used as a loading control. The arrow indicates the cleaved form of PARP, while the upper band corresponds to total PARP protein. Results are presented as the percentage (B, C) or percent fold change (A) relative to control cells (CTRL). All data are expressed as the mean ± SD from at least three independent experiments (A, B, C). Pictures are from a single experiment, representative of three independent experiments (A, C, D). P-values from a Student t-test are represented as $*p < 0.05$; $**p < 0.01$; $***p < 0.001$.

Figure S3

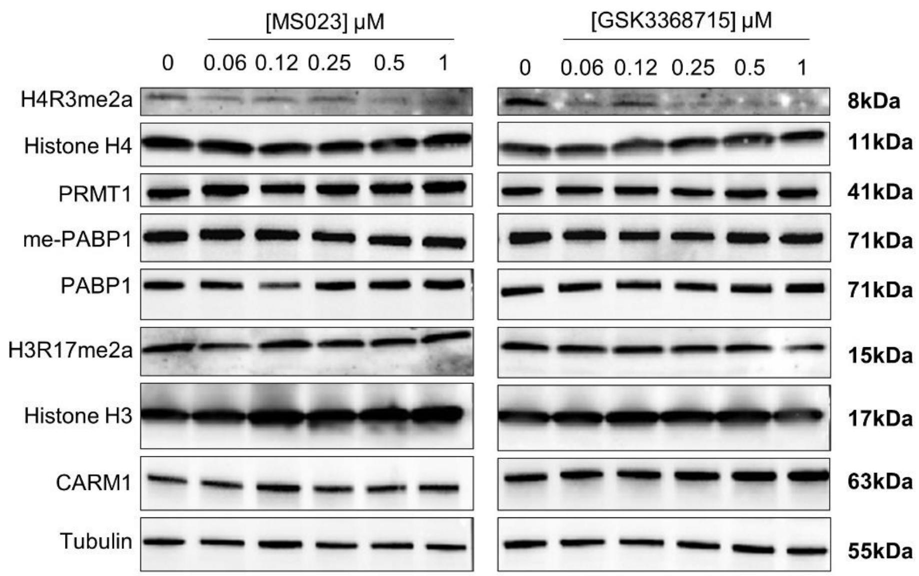

Figure S3. Type I PRMT inhibitors decrease PRMT1 but not CARM1 and PRMT6 activity under the tested conditions.

MDA-MB-468 cells were treated with varying concentrations of MS023 or GSK3368715 for 48h. PRMT1 inhibition was assessed by western blotting using anti-H4R3me2a antibody. Methylation of PABP1 (me-PABP1) was used to measure CARM1 activity and histone H3 methylation (H3R17me2a) to assess CARM1 and PRMT6 activities. Anti-PABP1, histone H3, CARM1, and tubulin antibodies were used as loading controls.

Figure S4

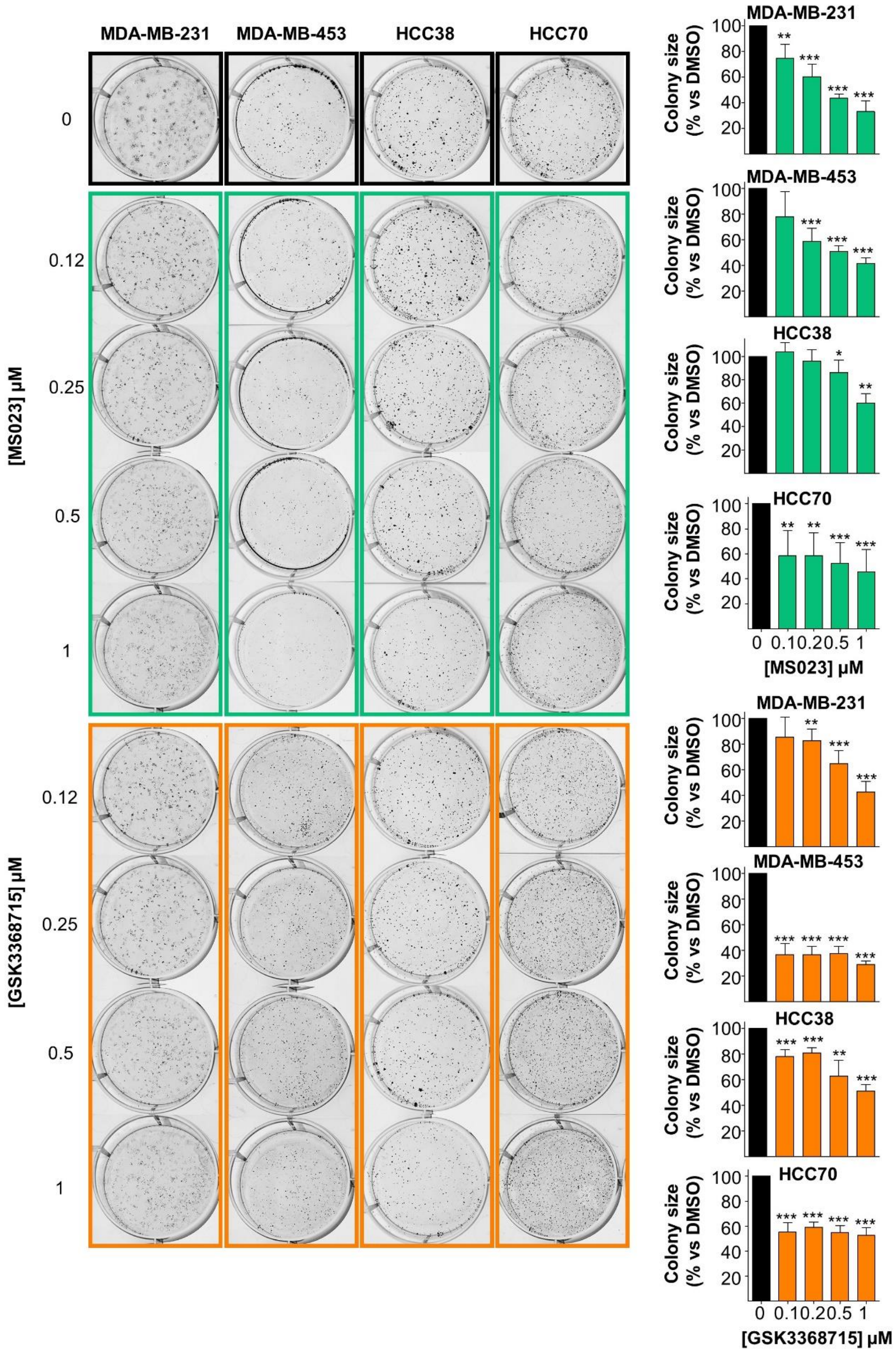

MDA-MB-231
MDA-MB-453
HCC38
HCC70
0
0.12
0.25
0.5
1
[MS023] µM
[GSK3368715] µM
Colony size (% vs DMSO)
0 0.1 0.2 0.5 1

Figure S4. Type I PRMT inhibitors decrease colony size in TNBC cells.
MDA-MB-231, MDA-MB-453, HCC38, and HCC70 cells were cultured on plastic for 7 (MDA-MB-231) or 14 (MDA-MB-453, HCC38, and HCC70) days after MS023 (green) or GSK3368715 (orange) treatment. The quantification of colony size is expressed as a percentage relative to DMSO-treated cells (black), represented as the mean ± SD from two independent experiments performed in triplicates. P-values are from a Student t-test and represented as *$p < 0.05$; **$p < 0.01$; ***$p < 0.001$.

Figure S5

A

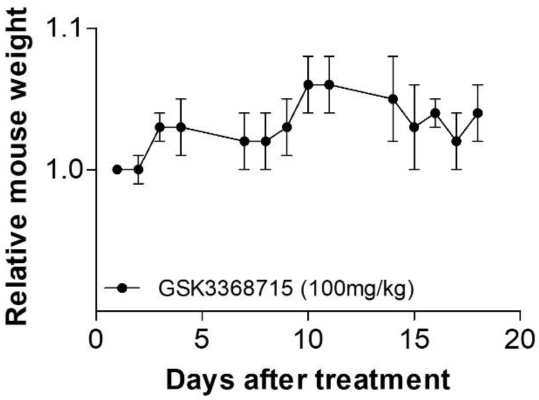

B

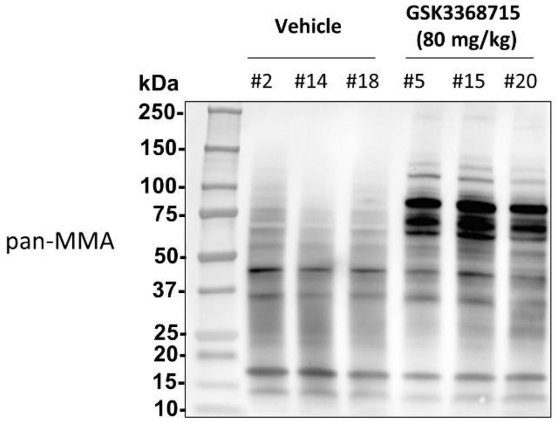

Figure S5. GSK3368715 treatment shows no toxicity and increases global monomethylation in mice.

A, GSK3368715 treatment is not toxic for mice at the tested dose. GSK3368715 was administered in Swiss-nude mice (n=3) at 100 mg/kg per-os, once daily for 18 days. Treatment was not associated with any mortality or body weight loss during this period.

B, GSK3368715 treatment (80 mg/kg) increases global monomethylation, *in vivo***.** Total monomethylation was detected by western blotting using anti-pan monomethylated (pan-MMA) antibodies in the tumors excised from 3 vehicle (#2, #14, #18)- or GSK3368715 (#5, #15, #20)-treated mice at the end of the experiment.

Figure S6

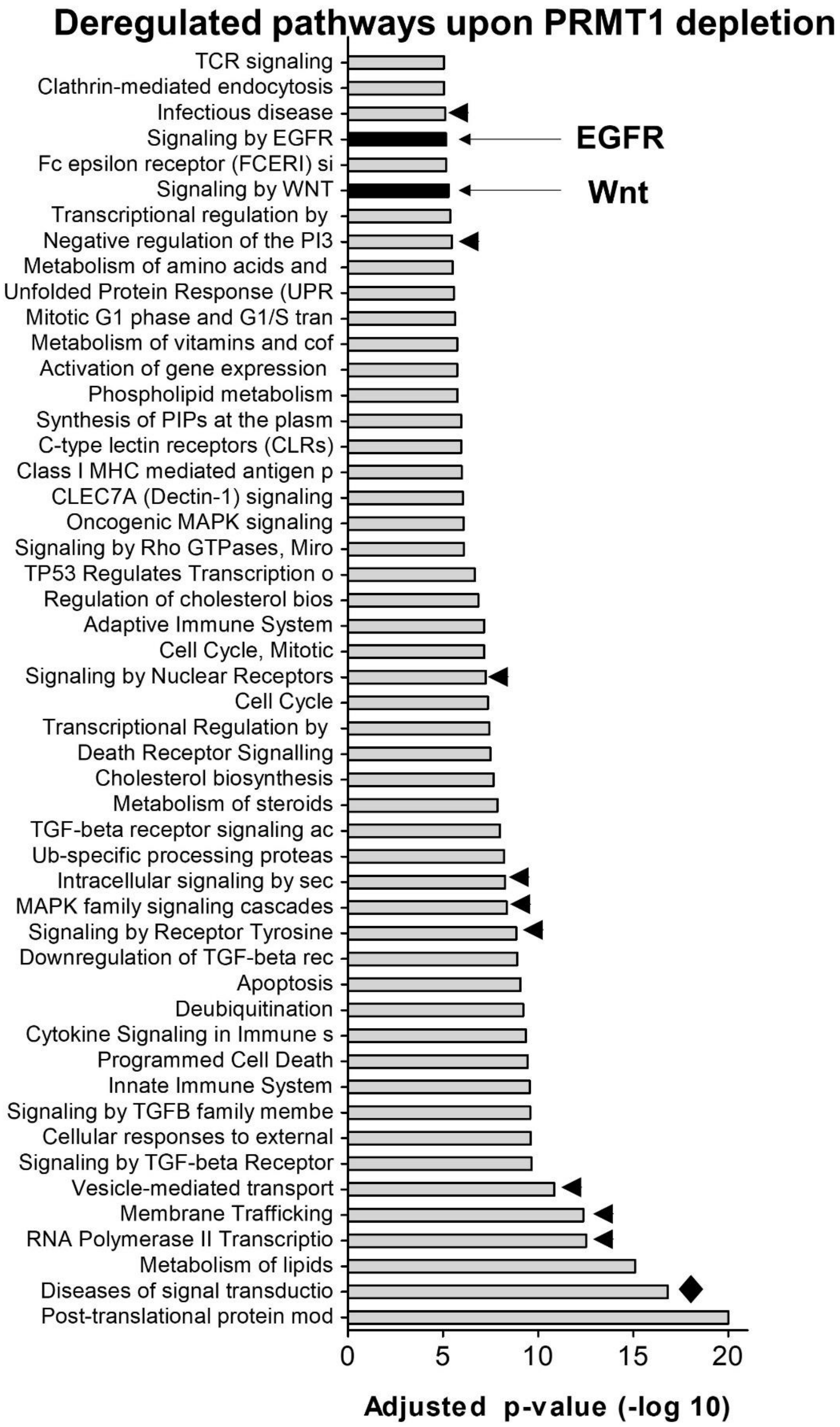
Deregulated pathways upon PRMT1 depletion
TCR signaling
Clathrin-mediated endocytosis
Infectious disease
Signaling by EGFR
Fc epsilon receptor (FCERI) si
Signaling by WNT
Transcriptional regulation by
Negative regulation of the PI3
Metabolism of amino acids and
Unfolded Protein Response (UPR
Mitotic G1 phase and G1/S tran
Metabolism of vitamins and cof
Activation of gene expression
Phospholipid metabolism
Synthesis of PIPs at the plasm
C-type lectin receptors (CLRs)
Class I MHC mediated antigen p
CLEC7A (Dectin-1) signaling
Oncogenic MAPK signaling
Signaling by Rho GTPases, Miro
TP53 Regulates Transcription o
Regulation of cholesterol bios
Adaptive Immune System
Cell Cycle, Mitotic
Signaling by Nuclear Receptors
Cell Cycle
Transcriptional Regulation by
Death Receptor Signalling
Cholesterol biosynthesis
Metabolism of steroids
TGF-beta receptor signaling ac
Ub-specific processing proteas
Intracellular signaling by sec
MAPK family signaling cascades
Signaling by Receptor Tyrosine
Downregulation of TGF-beta rec
Apoptosis
Deubiquitination
Cytokine Signaling in Immune s
Programmed Cell Death
Innate Immune System
Signaling by TGFB family membe
Cellular responses to external
Signaling by TGF-beta Receptor
Vesicle-mediated transport
Membrane Trafficking
RNA Polymerase II Transcriptio
Metabolism of lipids
Diseases of signal transductio
Post-translational protein mod
EGFR
Wnt
0
5
10
15
20
Adjusted p-value (-log 10)

Figure S6. PRMT1 regulates EGFR and Wnt signaling pathways.

RNA was extracted from MDA-MB-468 cells transfected with control or PRMT1 (#7, #8) siRNA for 24h and 48h, and analyzed by Affymetrix microarray. Gene enrichment pathway analysis, using the REACTOME database, was performed on the deregulated genes common to both PRMT1 siRNAs. The top 50 deregulated pathways ranked according to their significance (adjusted p-values) is shown. The EGFR and Wnt signalling pathways are highlighted in black. Arrowheads point to pathways including *EGFR*, and diamond points to pathways including *EGFR*, *LRP5* and *PORCN*.

Figure S7

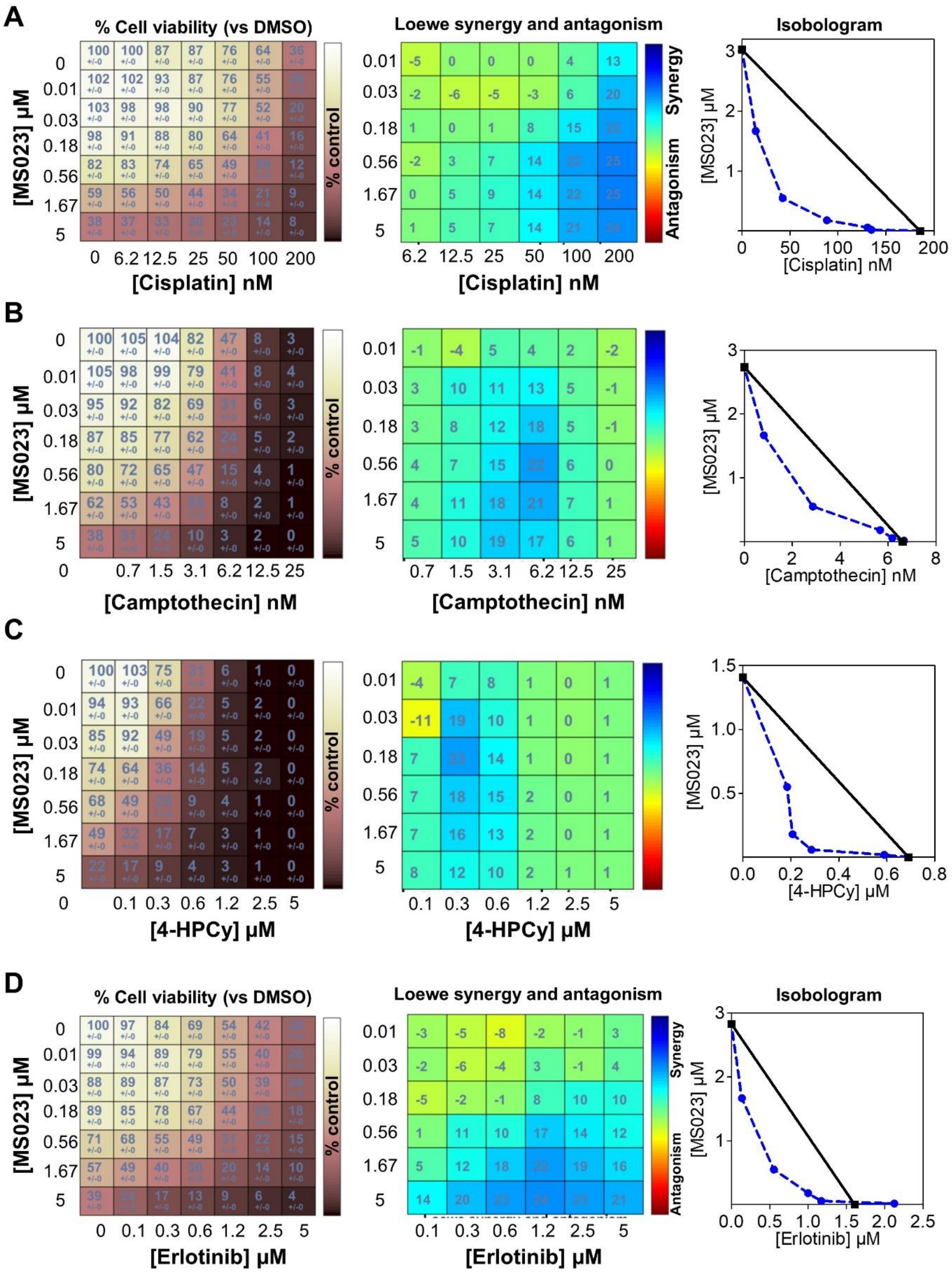
A
% Cell viability (vs DMSO)
Loewe synergy and antagonism
Isobologram
[MS023] µM
% control
Synergy
Antagonism
[Cisplatin] nM
B
[Camptothecin] nM
C
[4-HPCy] µM
D
[Erlotinib] µM

Figure S7. Synergistic interactions between MS023 (a type I PRMT inhibitor) and chemotherapies (A, B, C) or erlotinib (D).

MDA-MB-468 cells were seeded in 96-well plates, treated with the indicated drugs for 7 days (equivalent to 4 doubling times), and cell viability was measured by CellTiterGlo assay. MS023 was serially diluted three-fold and cisplatin (A), camptothecin (B), 4-hydroperoxy cyclophosphamide (4-HPCy; C), erlotinib (D) were serially diluted two-fold (concentrations indicated in the Figure). The drug interactions were calculated using the Loewe model on the Combenefit software. Cell viability (% compared to DMSO-treated cells, left panel), synergy matrix as calculated using the Loewe excess model (middle panel), and isobolograms (right panel) for each drug pair are indicated. Presented data are representative of three independent experiments.

Figure S8

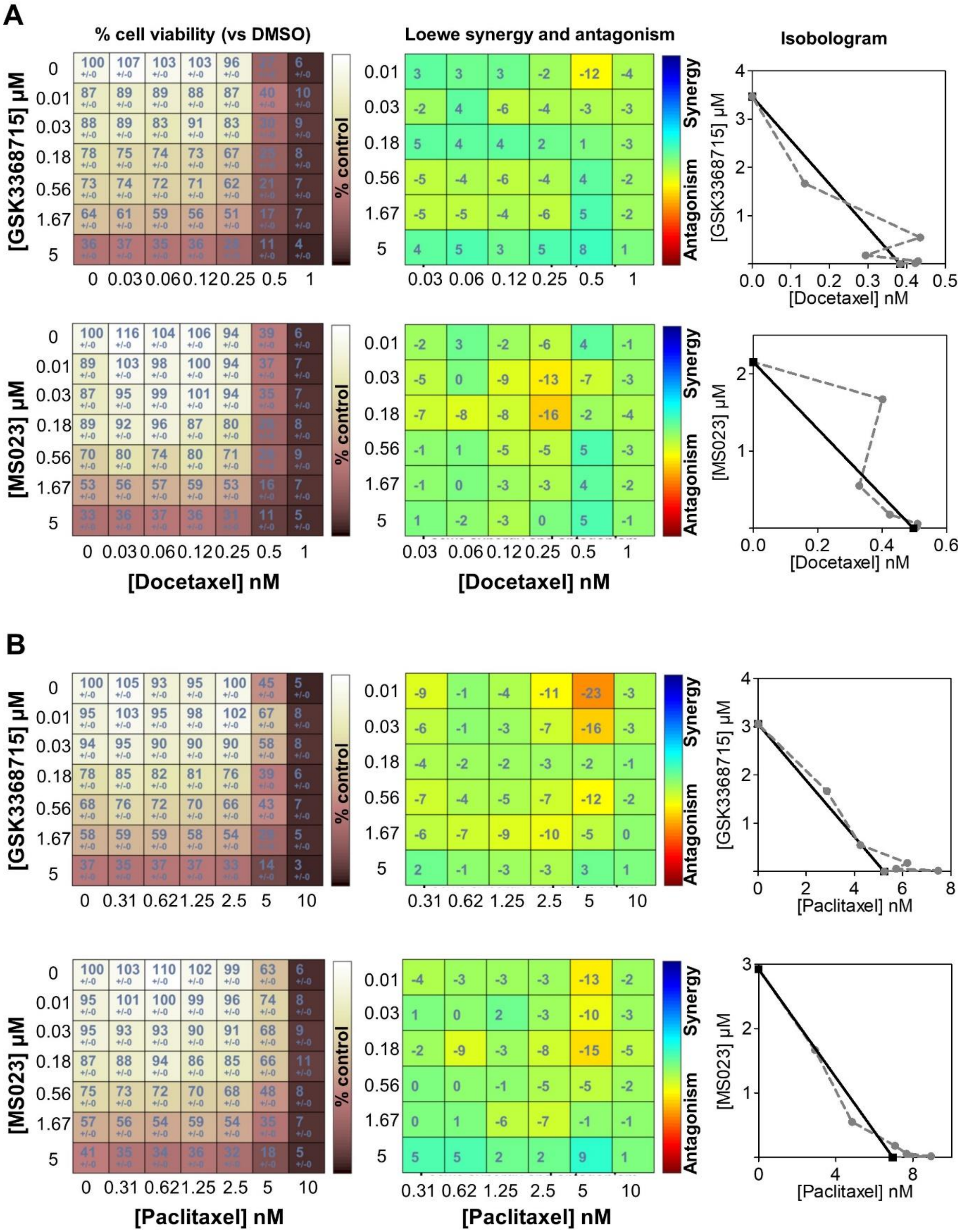
A
% cell viability (vs DMSO)
Loewe synergy and antagonism
Isobologram
[GSK3368715] µM
[MS023] µM
% control
Synergy
Antagonism
[Docetaxel] nM
B
[Paclitaxel] nM

Figure S8. Additive interactions between Type I PRMT inhibitors and taxanes.
A and B, MDA-MB-468 cells were seeded in 96-well plates, treated with the indicated drugs for 7 days (equivalent to 4 doubling times) and cell viability was measured by CellTiterglo assay. Type I PRMT inhibitors (MS023, GSK3368715) were serially diluted three-fold and docetaxel (A) or paclitaxel (B) were serially diluted two-fold (concentrations indicated in the figure). The drug interactions were calculated using the Loewe model on the Combenefit software. Cell viability (% compared to DMSO-treated cells, left panel), synergy matrix as calculated using the Loewe excess model (middle panel), and isobolograms (right panel) for each drug pair are indicated. Presented data are representative of three independent experiments.

Figure S9

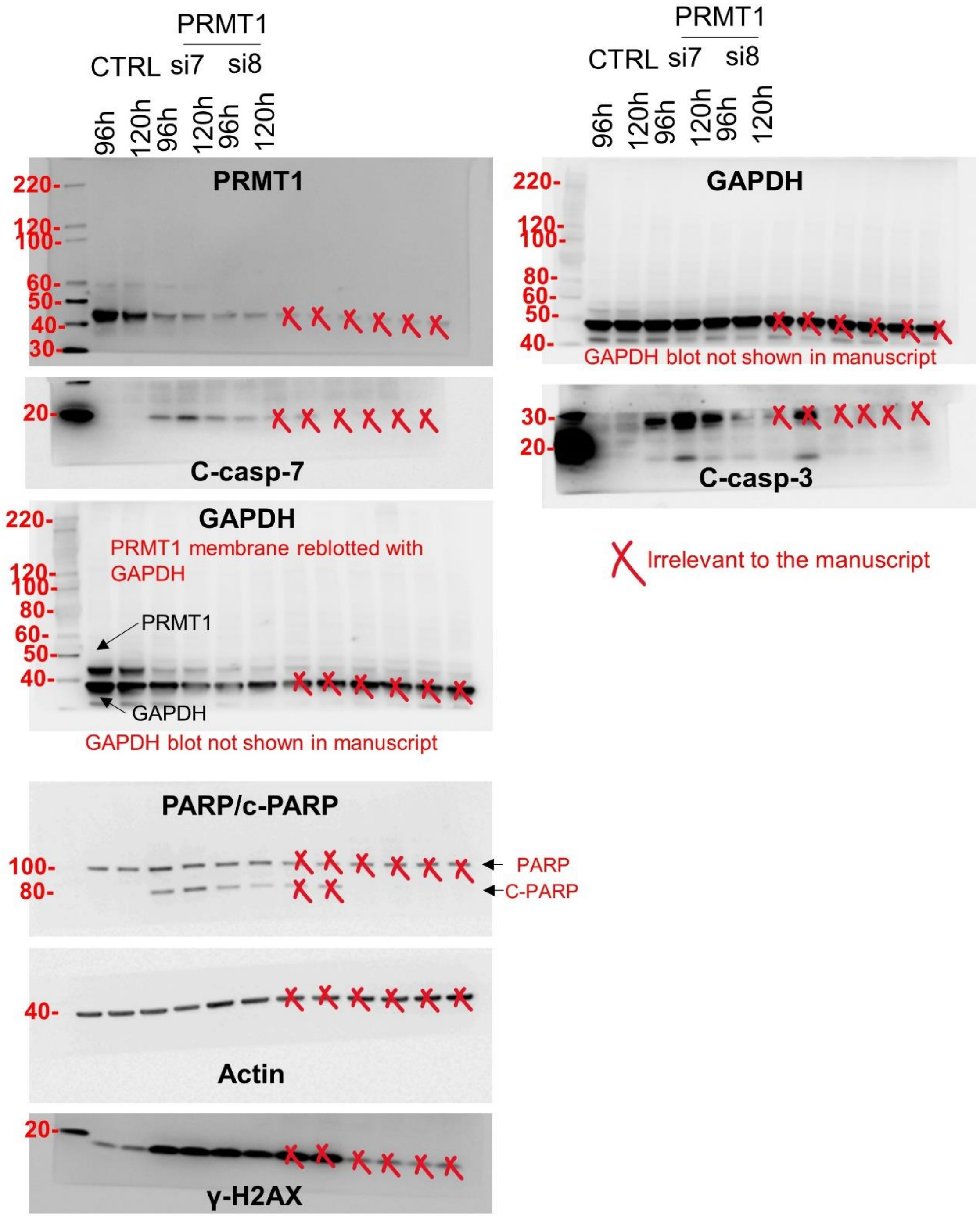

Figure S9. Uncropped original blots of Figure 2D.

Figure S10

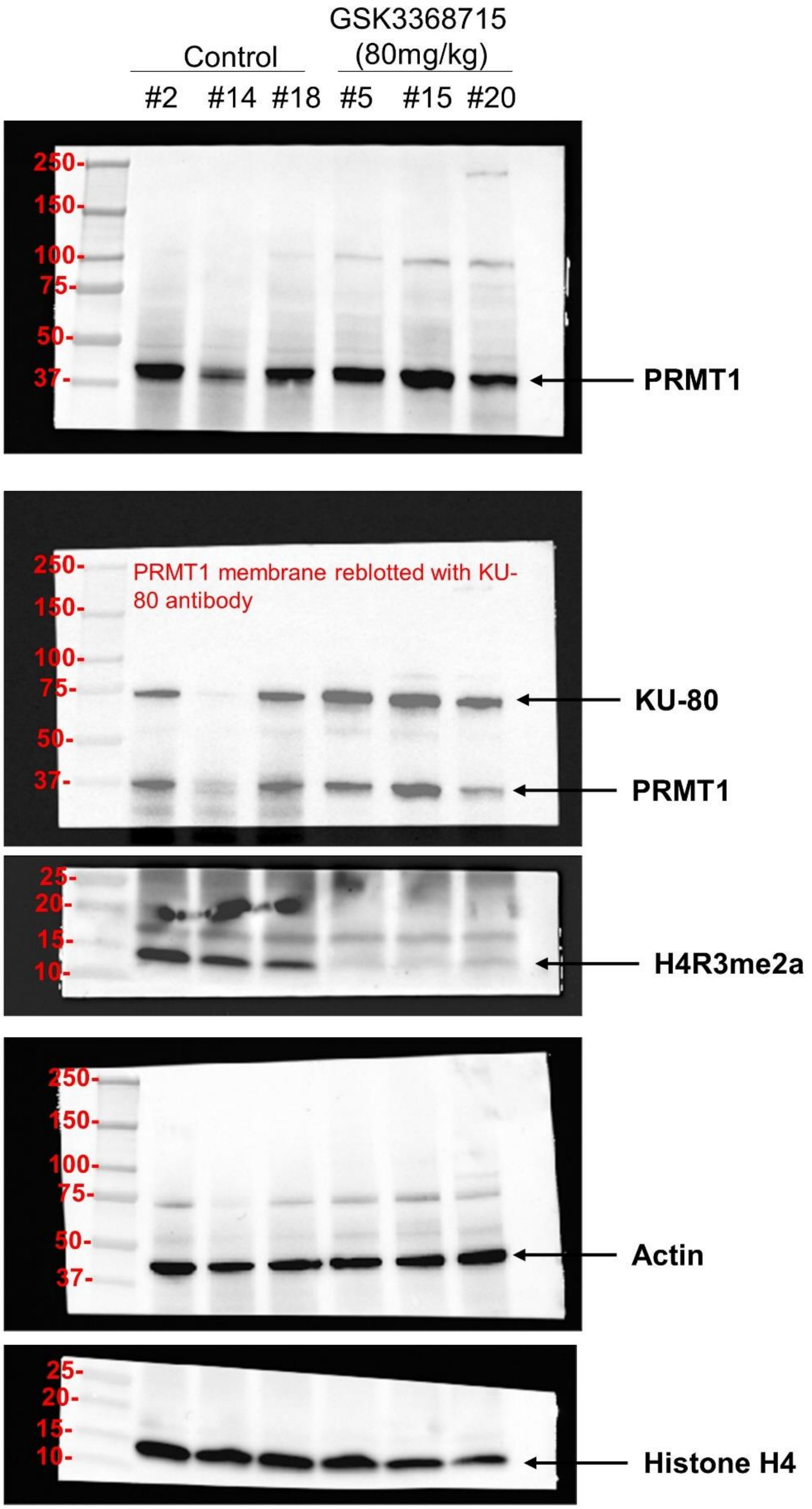

Figure S10. Uncropped original blots of Figure 3D.

Figure S11

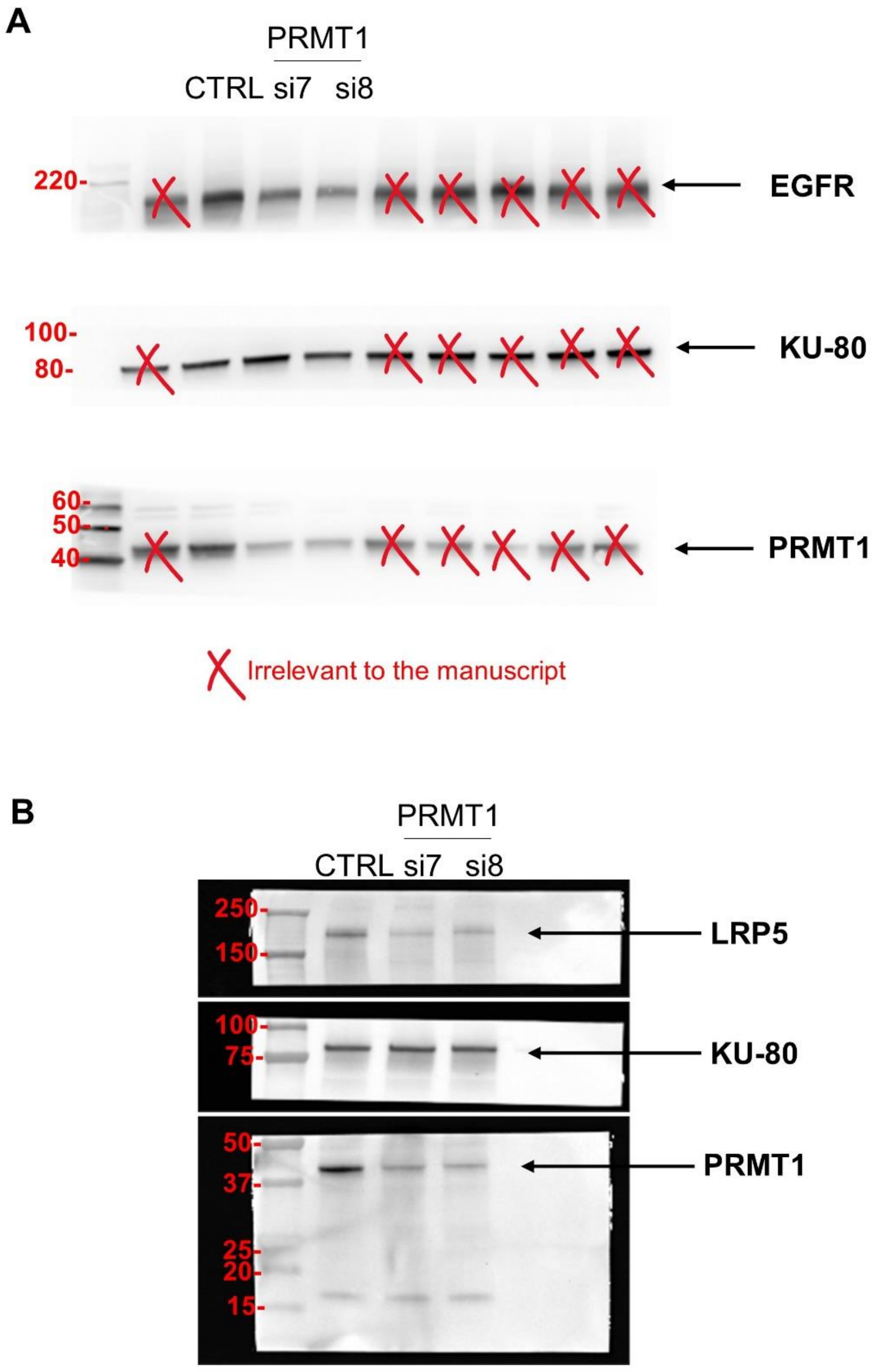

Figure S11. A, uncropped original blots of Figure 4D. B, uncropped original blots of Figure 4H.

Figure S12

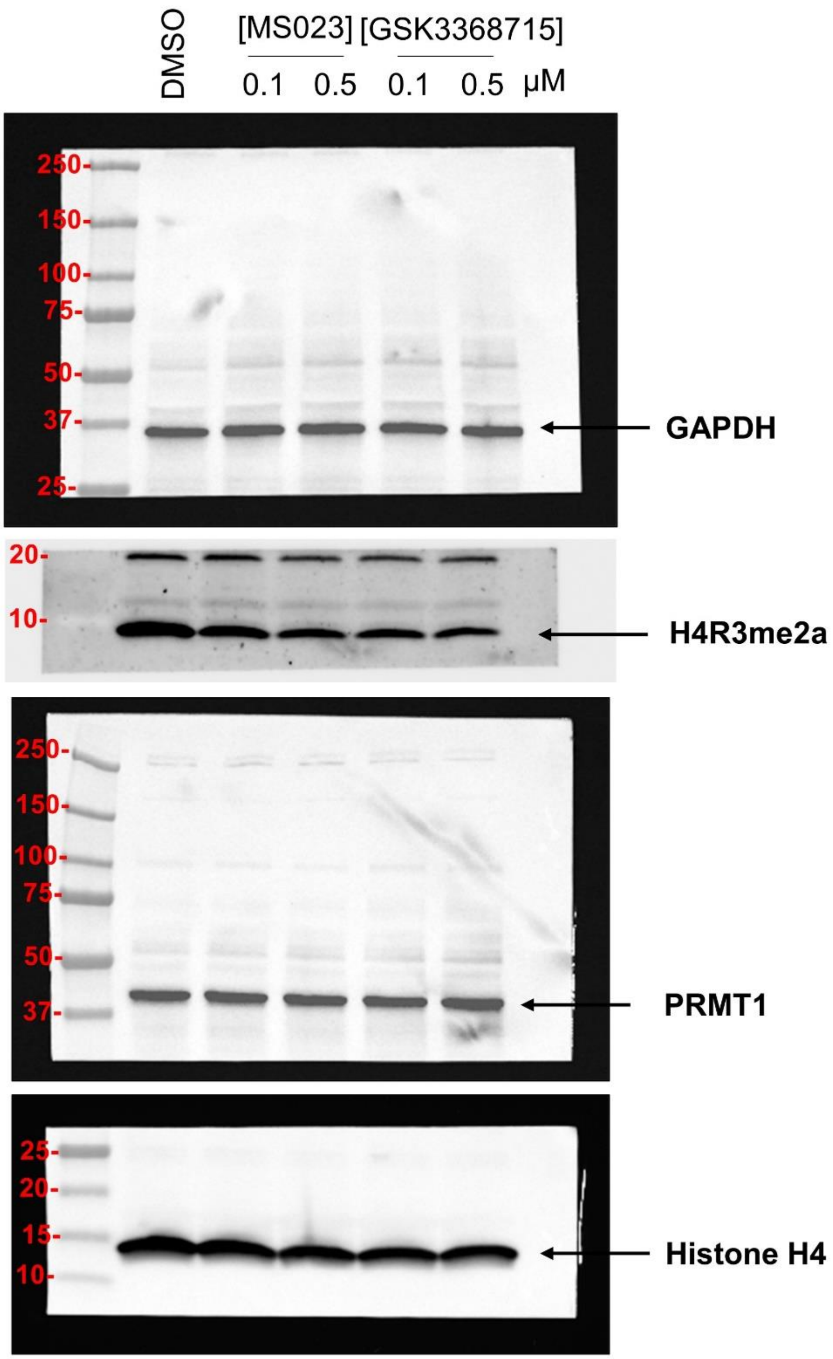

Figure S12. Uncropped original blots of Figure 5D.

Figure S13

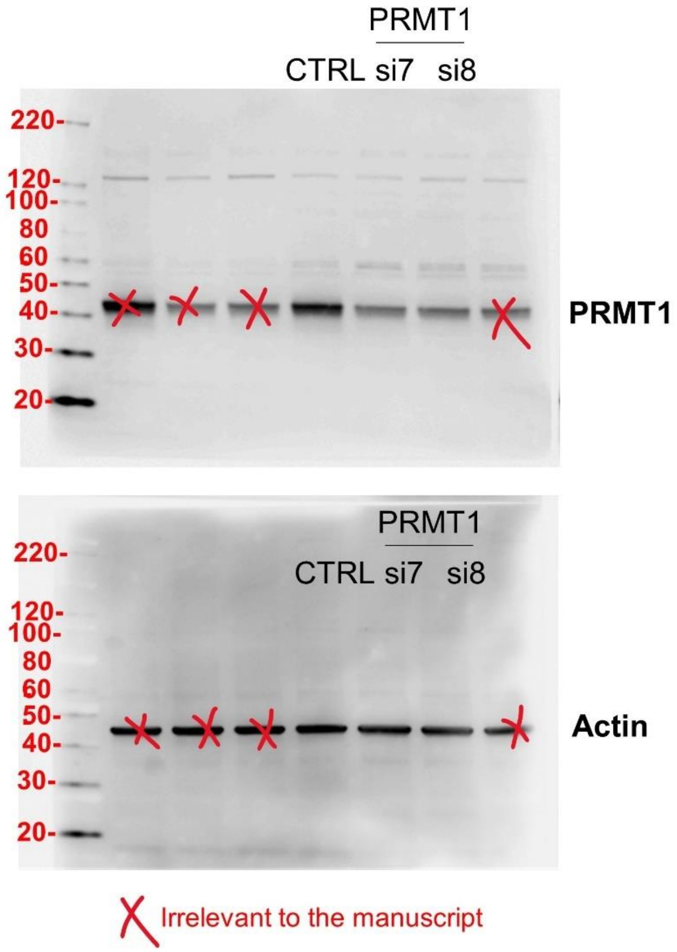

Figure S13. Uncropped original blots of Figure S1D.

Figure S14

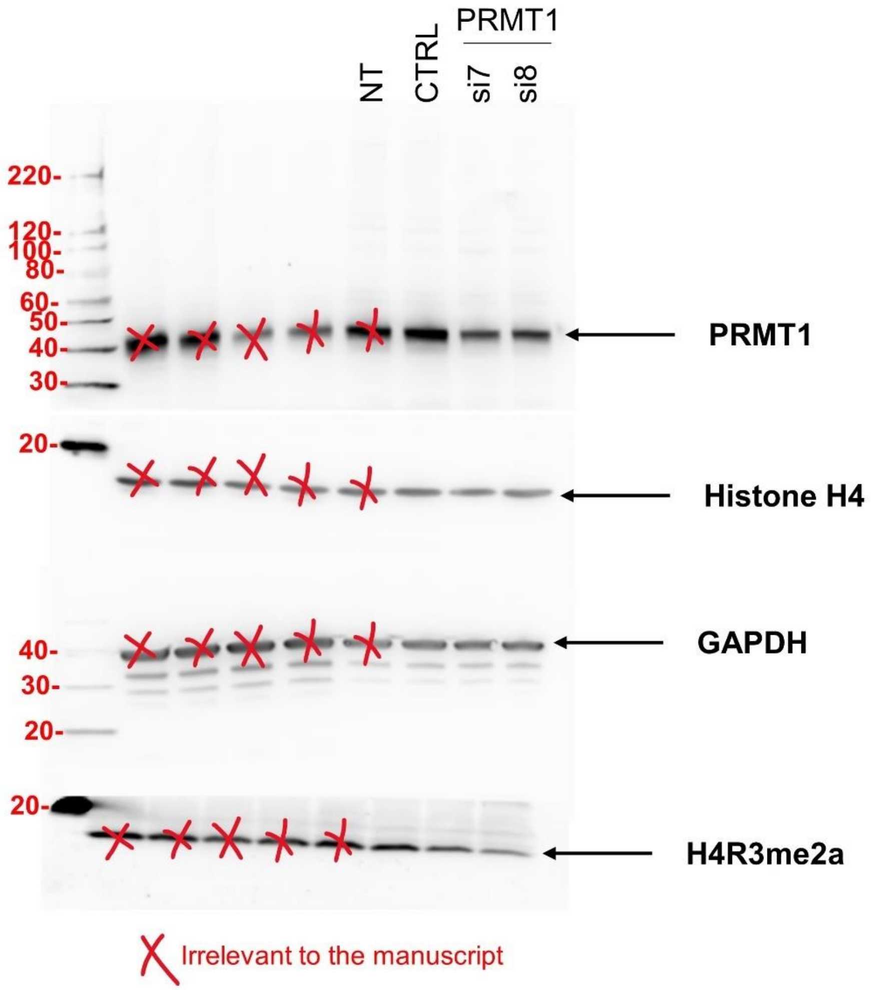

Figure S14. Uncropped original blots of Figure S2A. NT- non treated

Figure S15

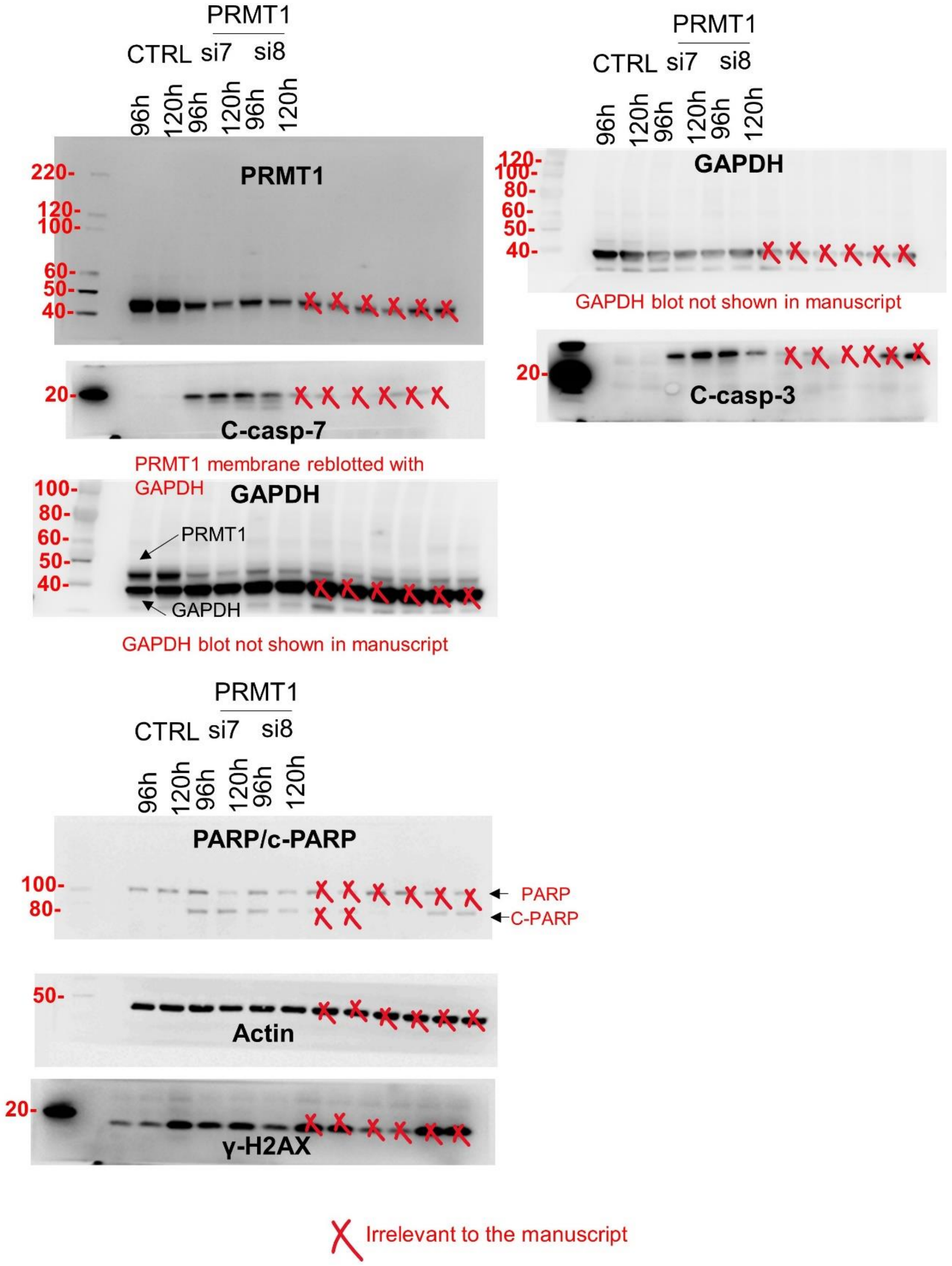

Figure S15. Uncropped original blots of Figure S2D for HCC70 cell line.

Figure S16

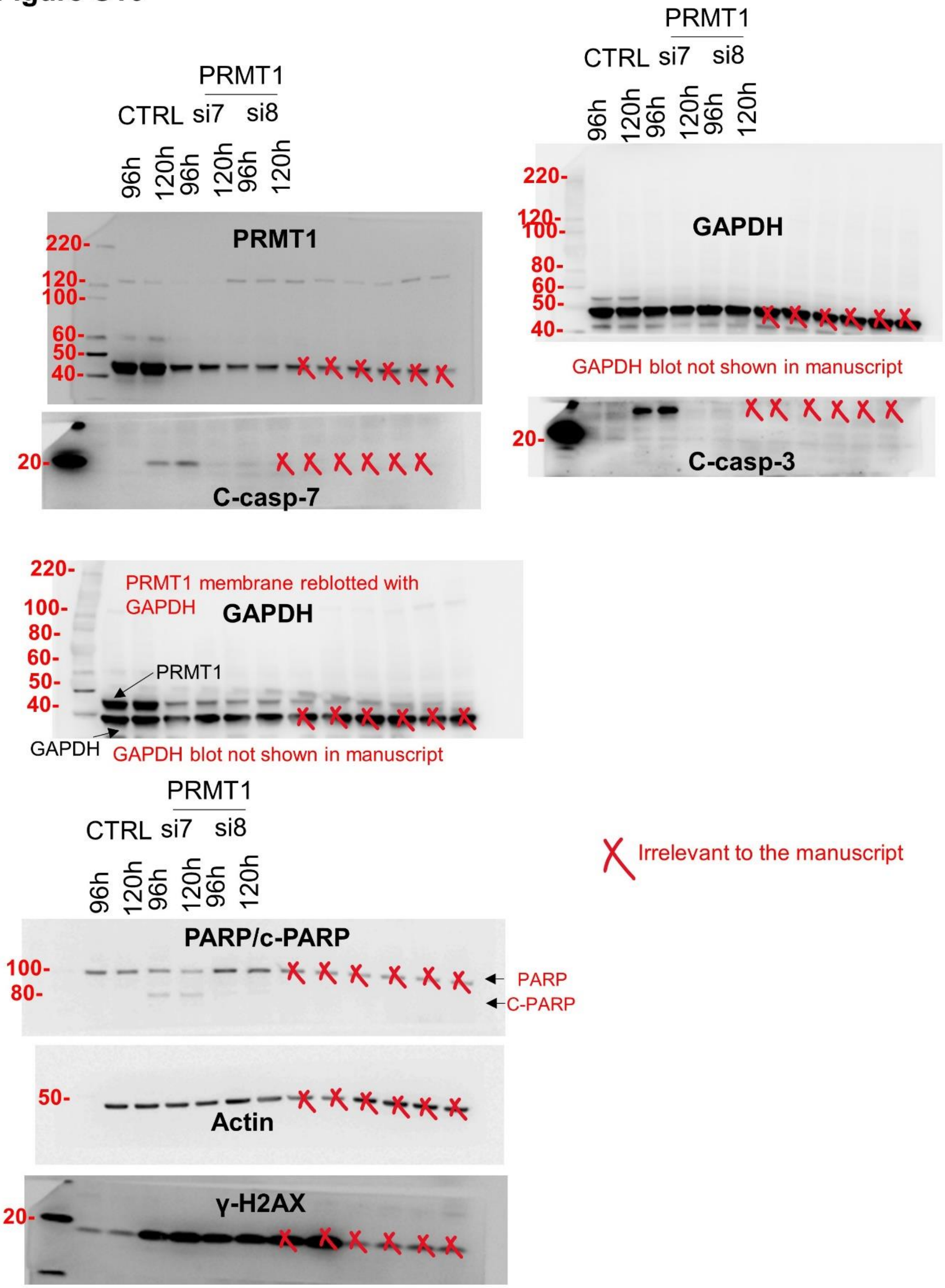

Figure S16. Uncropped original blots of Figure S2D for MDA-MB-231 cell line.

Figure S17

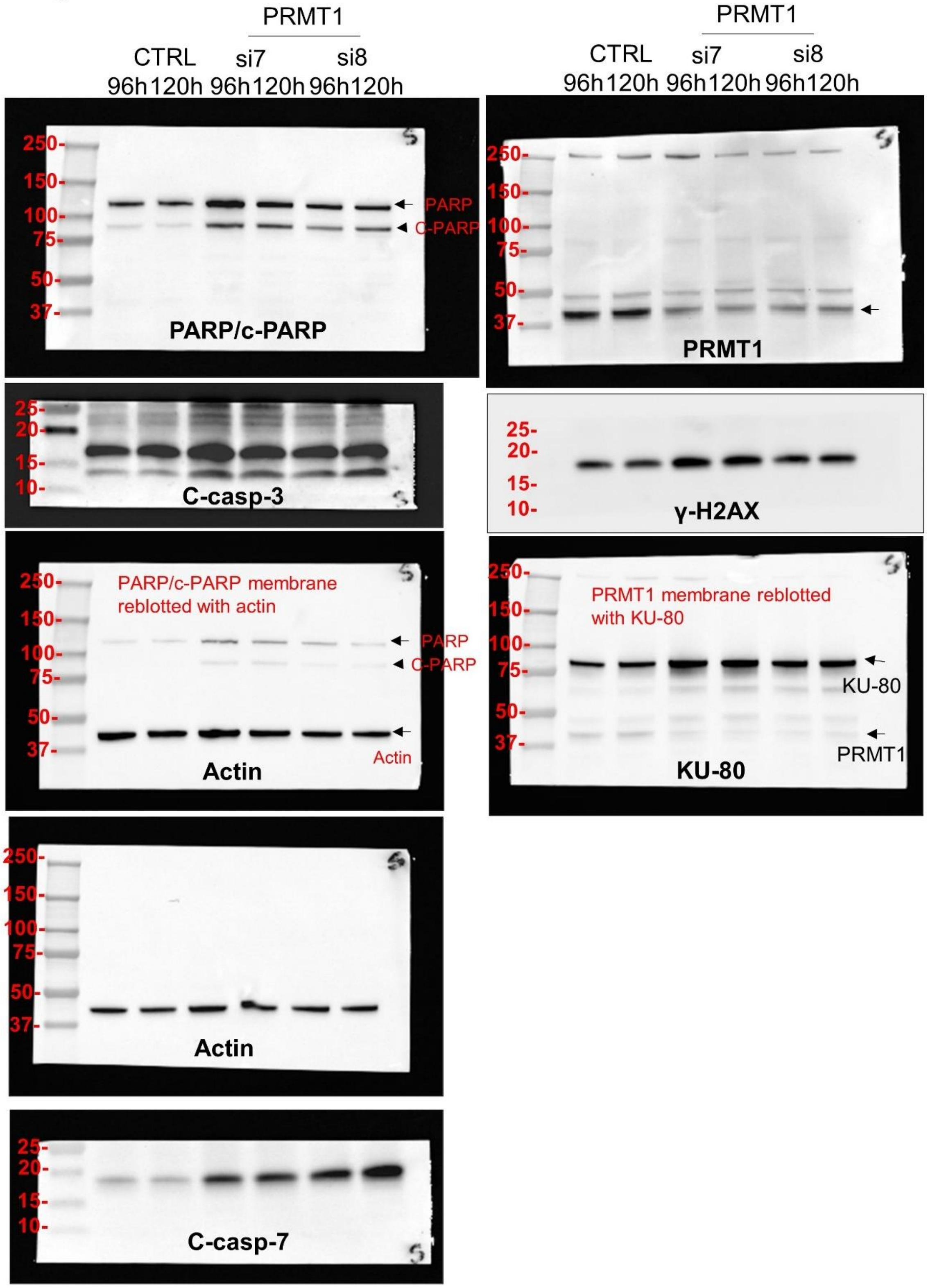

Figure S17. Uncropped original blots of Figure S2D for SKBr3 cell line.

Figure S18

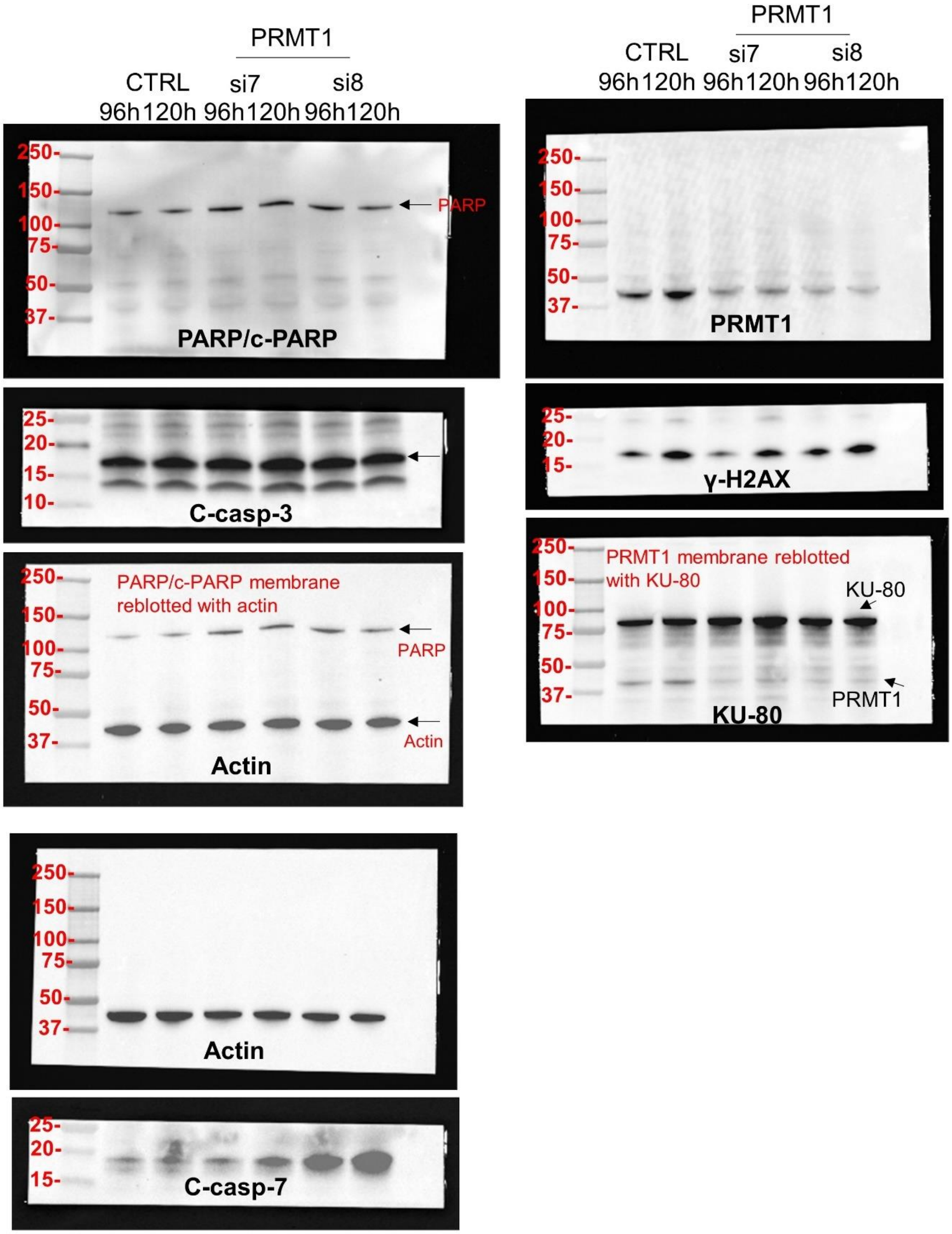

Figure S18. Uncropped original blots of Figure S2D for T47D cell line.

Figure S19

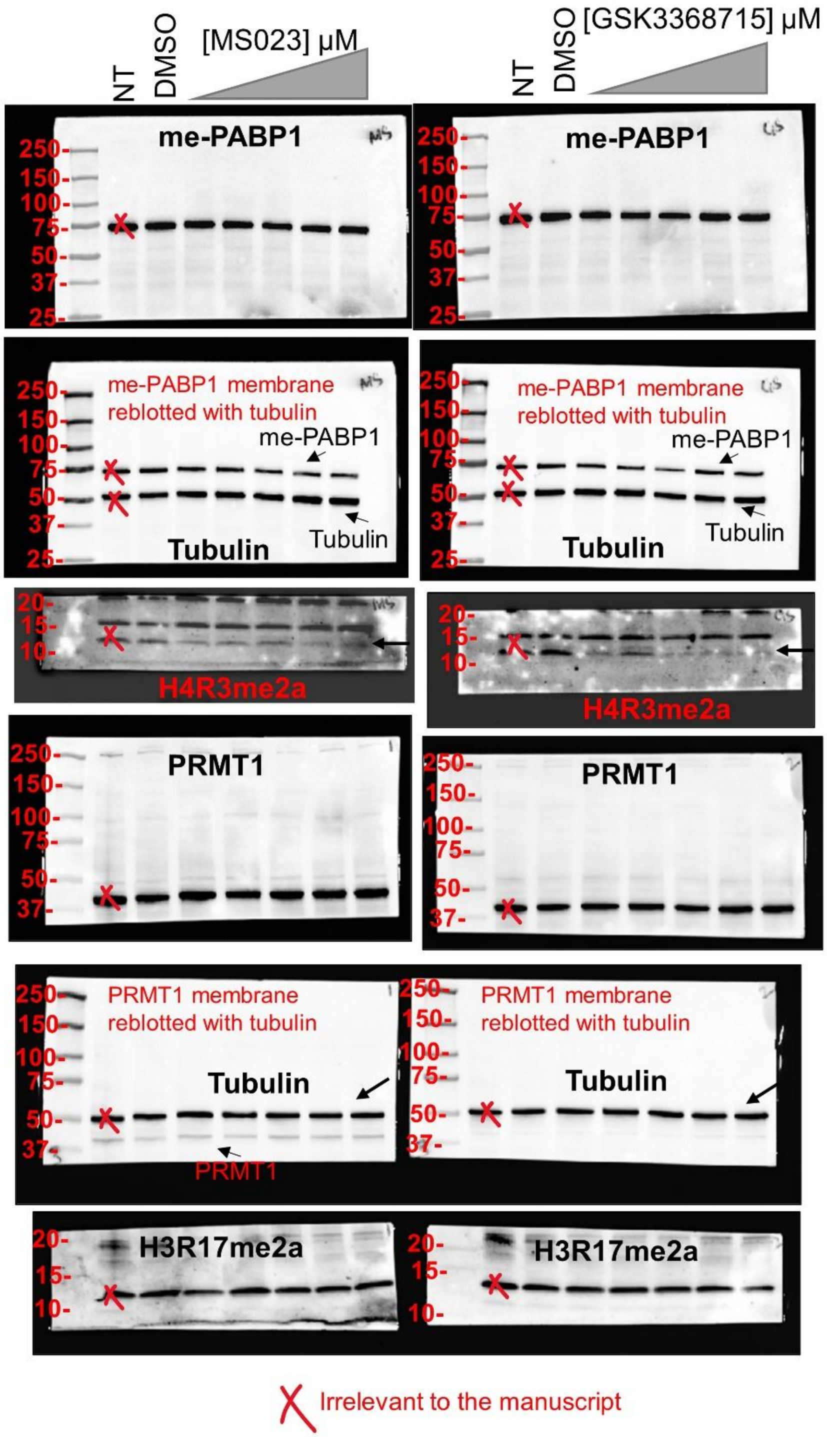

Figure S19. Uncropped original blots of Figure S3 (for remaining blots, see Figure S20). NT- non treated.

Figure S20

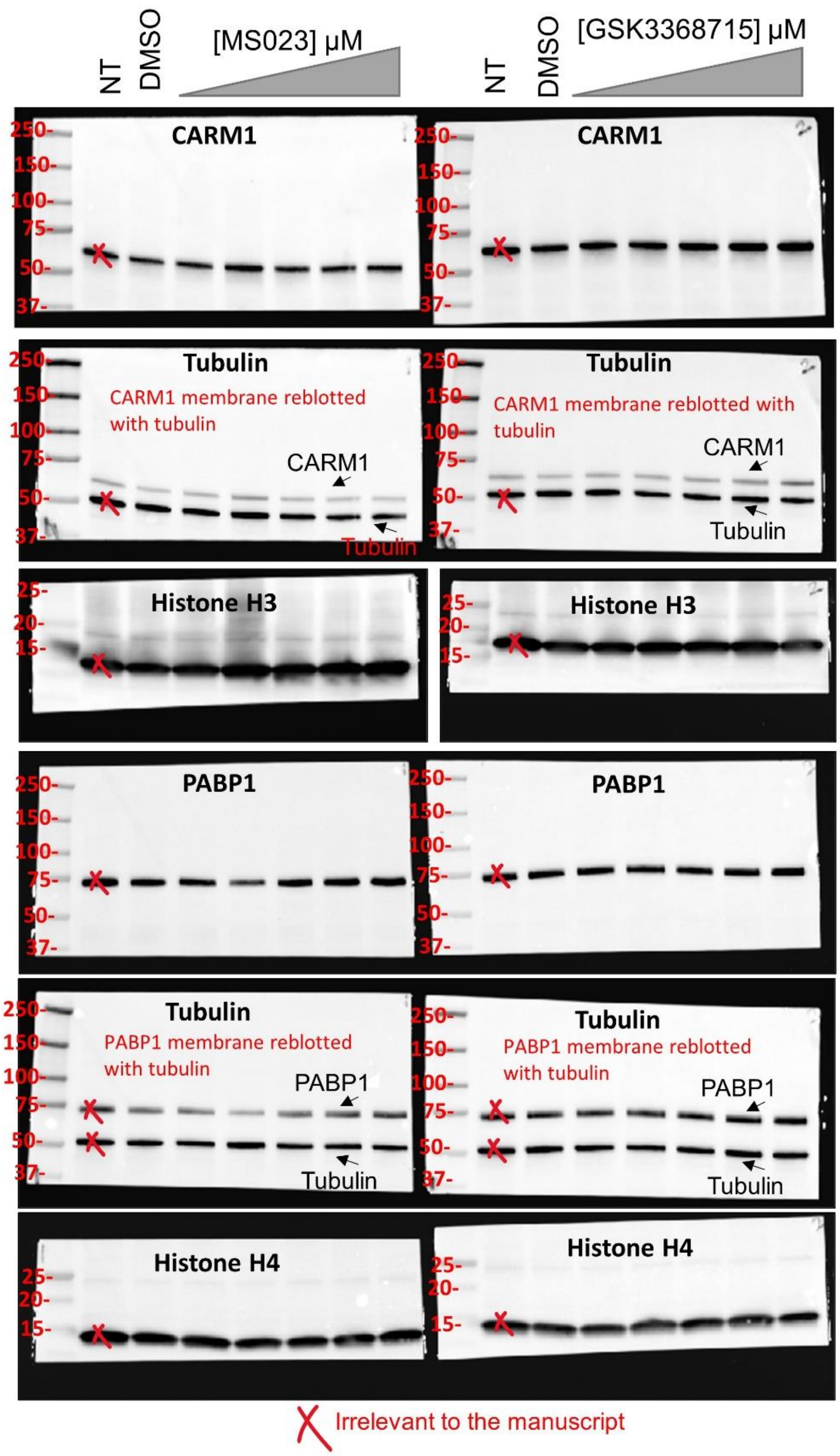

Figure S20. Remaining uncropped original blots of Figure S3. NT- non treated.

Figure S21

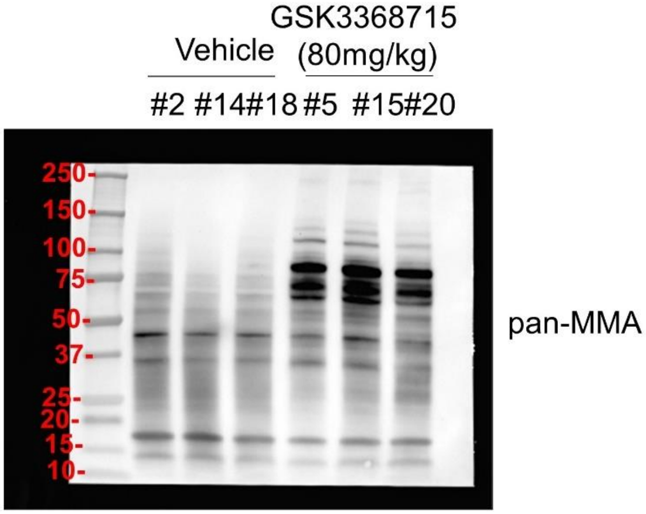

Figure S21. Uncropped original blot of Figure S5B.

Table S1. (separate file)
Antibodies, primers, siRNAs and drugs

Table S2. (separate file)
Differentially expressed genes in PRMT1-depleted MDA-MB-468 cells

Table S3. (separate file)
PRMT1 DNA copy number gain and loss in the curie cohort
